# Beneficial effects of ginkgetin on improving nonalcoholic steatohepatitis characterized by bulk and single-cell RNA sequencing analysis

**DOI:** 10.3389/fphar.2023.1267445

**Published:** 2023-10-04

**Authors:** Chaoyang Wang, Yaowei Bai, Tongqiang Li, Jiacheng Liu, Yingliang Wang, Shuguang Ju, Wei Yao, Bin Xiong, Guofeng Zhou

**Affiliations:** ^1^ Department of Radiology, Union Hospital, Tongji Medical College, Huazhong University of Science and Technology, Wuhan, China; ^2^ Department of Interventional Radiology, The First Affiliated Hospital of Guangzhou Medical University, Guangzhou, China

**Keywords:** ginkgetin, nonalcoholic steatohepatitis, NASH, bulk, single cell, RNA sequecing

## Abstract

**Background and aims:** Nonalcoholic steatohepatitis (NASH) has become one of the major causes of cirrhosis and liver failure. However, there are currently no approved medications for managing NASH. Our study was designed to assess the effects of ginkgetin on NASH and the involved mechanisms.

**Methods:** We constructed a mouse model of NASH by high-fat diet for 24 weeks. The effects of ginkgetin on NASH were evaluated by histological study, Western blot, and biochemical analysis. RNA Sequencing (RNA-Seq) analysis was used to investigate the alteration in gene expression and signaling pathways at bulk and single-cell levels.

**Results:** Administration of ginkgetin resulted in a marked improvement in hepatic lipid accumulation, inflammation, and fibrosis in the NASH model. And these results were supported by bulk RNA-Seq analysis, in which the related signaling pathways and gene expression were markedly downregulated. Furthermore, single-cell RNA-Seq (scRNA-Seq) analysis revealed that the effects of ginkgetin on NASH were associated with the reprogramming of macrophages, hepatic stellate cells, and endothelial cells. Especially, ginkgetin induced a marked decrease in macrophages and a shift from pro-inflammatory to anti-inflammatory phenotype in NASH mice. And the NASH-associated macrophages (NAMs), which emerge during NASH, were also significantly downregulated by ginkgetin.

**Conclusion:** Ginkgetin exhibits beneficial effects on improving NASH, supported by bulk and single-cell RNA-Seq. Our study may promote pharmacological therapy for NASH and raise the existent understanding of NASH.

## Introduction

Nonalcoholic fatty liver disease (NAFLD), mainly caused by overnutrition or genetic defects, has been a major health concern worldwide, with a prevalence of 22.1%–28.6% (Younossi et al., 2018; Powell et al., 2021). NASH is an advanced stage of NAFLD, characterized by hepatocyte steatosis and ballooning, hepatic inflammation, and apoptotic body (Schuster et al., 2018). And NASH has a high risk of developing liver fibrosis, which can potentially advance to liver cirrhosis or cancer (Schuppan et al., 2018; Pinter et al., 2023). In addition, NASH is closely associated with cardiovascular diseases (Cai et al., 2019). Diet control and enhanced exercise are recommended for NASH treatment, but with limited effectiveness (Chalasani et al., 2018; Akuta et al., 2023). Therefore, investigating effective pharmaceutical therapy is currently necessary and urgent.

Traditional Chinese medicine has greatly contributed to the Chinese people’s health over the past thousands of years, especially in epidemic and metabolic diseases (Tu, 2016; Ma et al., 2020; Zhu et al., 2020; Huang et al., 2021; Chen et al., 2022). In recent years, growing studies have been performed to investigate the mechanism of traditional Chinese medicine using modern medical methods and achieved excellent results (Wang et al., 2018; Li et al., 2020; Wang et al., 2021). Ginkgetin is a compound extracted from Ginkgo biloba leaves and exerts anti-inflammatory and anti-tumor activities (Zhang et al., 2017; Adnan et al., 2020; Lou et al., 2021; Menezes and Diederich, 2021). Cho et al. (2019)reported that ginkgetin inhibits adipogenesis by regulating STAT5/PPARγ/CEBPα signaling. Wu et al. (2022) reported that ginkgetin ameliorates cardiomyopathy caused by obesity through Nrf2/ARE signaling. However, whether ginkgetin has activity against NASH is still unknown.

Bulk RNA sequencing (RNA-Seq) has been extensively utilized in experimental and translational research (Kuksin et al., 2021; Thind et al., 2021). However, bulk RNA-Seq failed to evaluate cell heterogeneity as it only measures the global expression. The emergence of single-cell RNA sequencing (scRNA-Seq) enables the investigation of cell heterogeneity at the single-cell level, which has been applied in NASH-related studies (Xiong et al., 2019; Hendrikx et al., 2022). In this study, we investigate the impacts of ginkgetin on NASH and the alteration in gene expression profile using bulk and scRNA-Seq analysis.

## Materials and methods

### Animal experiments

All animal experiments were conducted in accordance with NIH guidelines and were approved by the Ethics Committee of Tongji Medical College (Wuhan, China). Male C57BL/6J mice aged 8 weeks were purchased from Tongji Medical College and were provided controllable circumstances (a temperature of 20°C–22°C, 12-h light/dark cycle, and unlimited access to food and water). For a duration of 24 weeks, the mice were provided either a chow diet or a high-fat diet (HFD). Ginkgetin was purchased from Climax Biotech Co., Ltd. (Chengdu, China). Vehicle (DMSO) or ginkgetin (10 mg/kg/day) was administrated intragastrically in the last 8 weeks. The mice were separated into three groups: The chow diet group, the HFD + vehicle group, and the HFD + ginkgetin group.

Fasting body weight (FBW) of all the mice was measured every 4 weeks. Liver weight (LW) was measured after the mice were killed. The indicators related to liver function (the levels of alanine aminotransferase (ALT) and aspartate aminotransferase (AST) in the serum) and lipid metabolism (the levels of triglyceride and cholesterol in the serum and liver tissues) were measured by commercial kits (Servicebio Co., Ltd., Wuhan, China). The levels of TREM2 in the serum were measured by ELISA kits (Servicebio).

### Histological study

The liver samples were preserved in 4% phosphate-buffered paraformaldehyde once the mice were sacrificed. After being dehydrated, embedded in paraffin, the samples were sliced into sections of 5 μm thick. To assess liver fibrosis, the sections underwent staining with hematoxylin and eosin (HE) as well as Sirius red. Lipid accumulation in the liver was assessed by staining frozen sections with Oil red O. The images were acquired through light microscopy (Nikon, Japan). For immunofluorescence, the sections were incubated consecutively with primary and secondary antibodies and 4’,6-diamidino-2-phenylindole (DAPI). After quenching tissue autofluorescence, the images were acquired using fluorescent microscopy (Nikon). Supplementary Table S1 displayed the antibodies for immunofluorescence.

### Western blot

Equal proteins obtained from liver tissues were subjected to gel electrophoresis and subsequently transferred onto polyvinylidene fluoride (PVDF) membranes. Afterward, the membranes were exposed to primary and secondary antibodies. The protein bands were detected by a chemiluminescence system (Clinix Co., Ltd., China). Supplementary Table S1 displayed the antibodies for Western blot.

### Scanning electron microscopy (SEM)

The liver tissues were put into the electron microscope fixation solution (Servicebio) immediately after isolation and cut into small blocks about 2 mm × 2 mm × 2 mm. The tissues were stored at room temperature for 4 h away from light and transferred to 4°C for storage. After dehydration, drying, and gold-coated treatment, the prepared specimens were detected by an SEM (SU8010, Hitachi, Japan).

### Bulk RNA-Seq analysis

We performed bulk RNA-Seq analysis on the liver tissues of mice with NASH treated with vehicle or ginkgetin (*n* = 3 per group). Trizol reagent (Servicebio) was utilized to extract total RNA from the liver tissues. mRNA with poly-A was isolated from total RNA and converted to complementary DNA (cDNA). RNA-Seq array was conducted on the Illumina Novaseq6000 platform. After getting the expression matrix, DESeq2 was used to calculate the difference between the two groups. Differently expressed genes (DEGs) were considered as fold change (FC) > 2 and adjust *p* < 0.05. Further analyses were performed using the corresponding R packages. The raw data of RNA-Seq were available in the GEO database with number GSE235797.

### scRNA-seq analysis

Liver non-parenchymal cells (LNPCs) were isolated from liver tissues of mice with NASH treated with vehicle or ginkgetin as previously described (Mederacke et al., 2015). Then LNPCs were used for scRNA-Seq by 10X Genomics Chromium system (*n* = 3 per group). Quality control was performed by Seurat (version 4.3.0), and only the single cells with the unique molecular identifier (UMI) between 250 and 5,000 and mitochondrial genes <5% were used for further analysis. The clusters were identified by marker genes according to the CellMarker database (Hu et al., 2023). Visual analysis was performed using R packages. The raw data of scRNA-Seq were available in the GEO database with number GSE235939.

### Macrophage polarization index (MPI)

MPI describes all the polarized states of macrophages. Based on scRNA-Seq, the MPI value of each macrophage was calculated according to a well-established model (M0: unstimulated; MI: stimulated with LPS and IFNγ; M2: stimulated with IL4 and IL13) (Li et al., 2019). A higher MPI value indicates that the macrophage is closer to a pro-inflammatory state of M1, and *vice versa*, a lower MPI value indicates an anti-inflammatory state of M2.

### Statistical analysis

The data were shown as mean ± SD and the difference was compared by *t*-test. Statistical analyses were carried out using SPSS 23.0 (IBM, United States). *p* < 0.05 was considered an indicator of statistical significance.

## Results

### Effects of ginkgetin on HFD-induced hepatic steatosis

**FIGURE 1:**
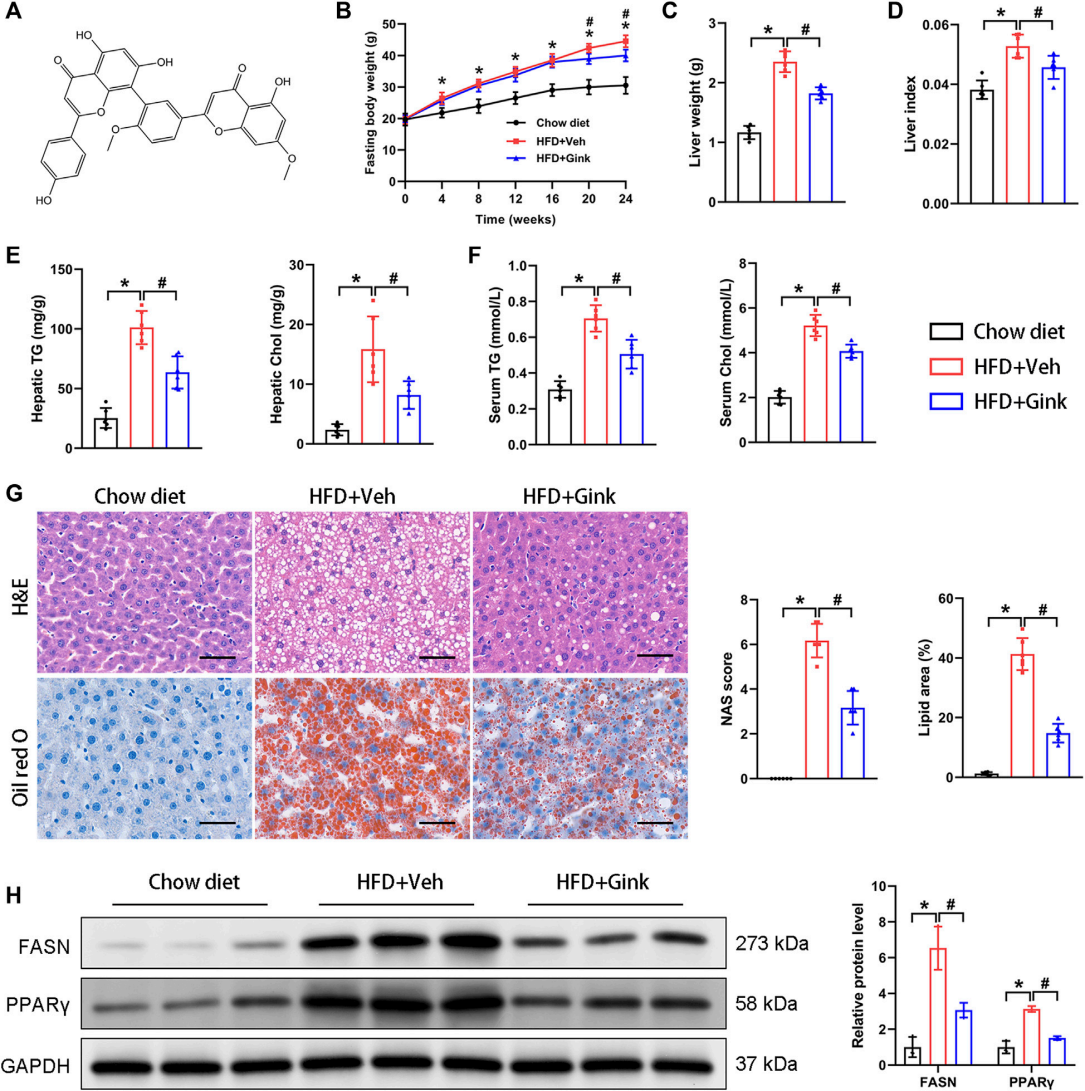
Effects of ginkgetin on HFD-induced hepatic steatosis. **(A)** The chemical formula of ginkgetin. **(B)** Changes in fasting body weight (FBW) over time in the chow diet, HFD + Veh, and HFD + Gink groups (*n* = 6). **(C)** Liver weight (LW) of the mice at the end of experiments (*n* = 6). **(D)** Liver index (FBW/LW) (*n* = 6). **(E)** Biochemical analysis of hepatic triglyceride (TG) and cholesterol (Chol) (*n* = 6). **(F)** Biochemical analysis of serum TG and Chol (*n* = 6). **(G)** HE and Oil red O staining of liver tissues (*n* = 6). Scale bar = 50 μm. **(H)** Western blot analysis of FASN and PPARγ (*n* = 3). *: *p* < 0.05, chow diet vs. HFD + Veh groups. #: *p* < 0.05, HFD + Veh vs. HFD + Gink groups.

The chemical formula of ginkgetin was shown in Figure 1A. We first evaluated the impacts of ginkgetin on hepatic steatosis in a NASH mouse model induced by HFD. Administration of HFD resulted in a clear increase in FBW, LW, and LW/FBW ratio, which was significantly improved by ginkgetin (Figures 1B–D). And ginkgetin-treated mice also showed reduced levels of hepatic and serum triglyceride and cholesterol (Figures 1E,F). Additionally, HE staining revealed that the hepatocyte steatosis and ballooning were markedly alleviated with reduced NAFLD activity score (NAS) after ginkgetin treatment. And Oil red O staining confirmed the reduced lipid accumulation in ginkgetin-treated mice (Figure 1G). Consistently, the protein expression of FASN and PPARγ, which are associated with lipid metabolism, were significantly downregulated by ginkgetin (Figure 1H).

### Effects of ginkgetin on HFD-induced hepatic inflammation and fibrosis

**FIGURE 2:**
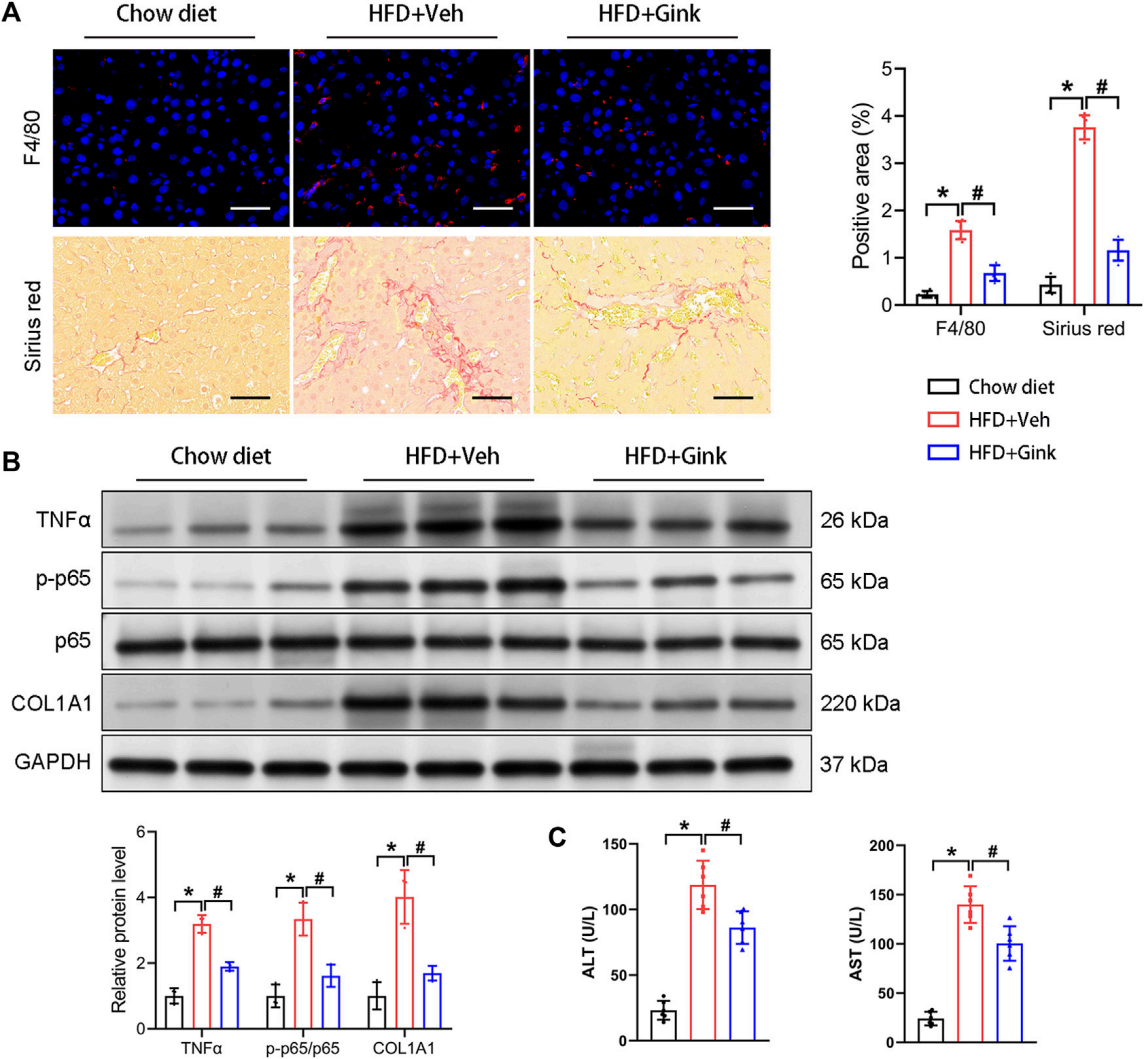
Effects of ginkgetin on HFD-induced hepatic inflammation and fibrosis. **(A)** F4/80 immunofluorescence and Sirus red staining of liver tissues in the chow diet, HFD + Veh, and HFD + Gink groups (*n* = 6). Scale bar = 50 μm. **(B)** Western blot analysis of proteins related to hepatic inflammation (TNFα and p65) and fibrosis (COL1A1) (*n* = 3). **(C)** The levels of ALT and AST in the three groups (*n* = 6). *: *p* < 0.05, chow diet vs. HFD + Veh groups. #: *p* < 0.05, HFD + Veh vs. HFD + Gink groups.

Immunofluorescence of F4/80 showed a marked increase in macrophage infiltration in mice with NASH, indicating enhanced hepatic inflammation, which was reduced in ginkgetin-treated mice (Figure 2A). And HFD-induced fibrosis, shown by Sirius red, was also improved after ginkgetin treatment (Figure 2A). Moreover, these histological alterations were supported by the level of proteins associated with inflammation (TNFα and p65) and fibrosis (COL1A1), which were overexpressed in NASH mice and reduced after ginkgetin treatment (Figure 2B). In addition, ginkgetin-treated mice showed improved liver function, shown by decreased ALT and AST (Figure 2C).

### Bulk RNA-Seq analysis of liver tissues from vehicle- and ginkgetin-treated mice with NASH

**FIGURE 3:**
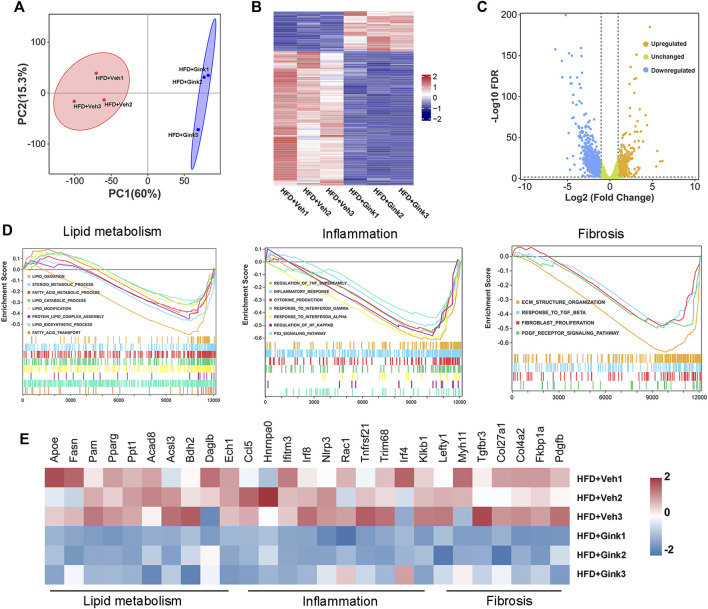
Bulk RNA-Seq analysis on liver tissues from NASH mice treated with vehicle and ginkgetin. **(A)** Principal component analysis between HFD + Veh and HFD + Gink groups (*n* = 3). **(B)** Heatmap of DEGs between the two groups. **(C)** Difference in gene expression between the two groups shown by volcano plot. **(D)** GSEA showed that the signals associated with lipid metabolism, hepatic inflammation and fibrosis were downregulated by ginkgetin. **(E)** Differences in gene expression based on GSEA.

To further investigate the process of ginkgetin-induced regression of NASH, we performed RNA-Seq analysis of liver tissues from NASH mice treated with vehicle or ginkgetin (*n* = 3 per group). The two groups could be clearly distinguished by principal component analysis (PCA) (Figure 3A). And 1,199 DEGs were observed, among which 406 were upregulated and 793 were downregulated by ginkgetin (Figures 3B, C). Gene set enrichment analysis (GSEA) showed that the signals associated with lipid metabolism, inflammation, and fibrosis were enriched and downregulated (Figure 3D; Supplementary Table S2). Consistently, the expression of related genes was downregulated by ginkgetin (Figure 3E).

### scRNA-seq analysis of LNPC from vehicle- and ginkgetin-treated mice with NASH

**FIGURE 4:**
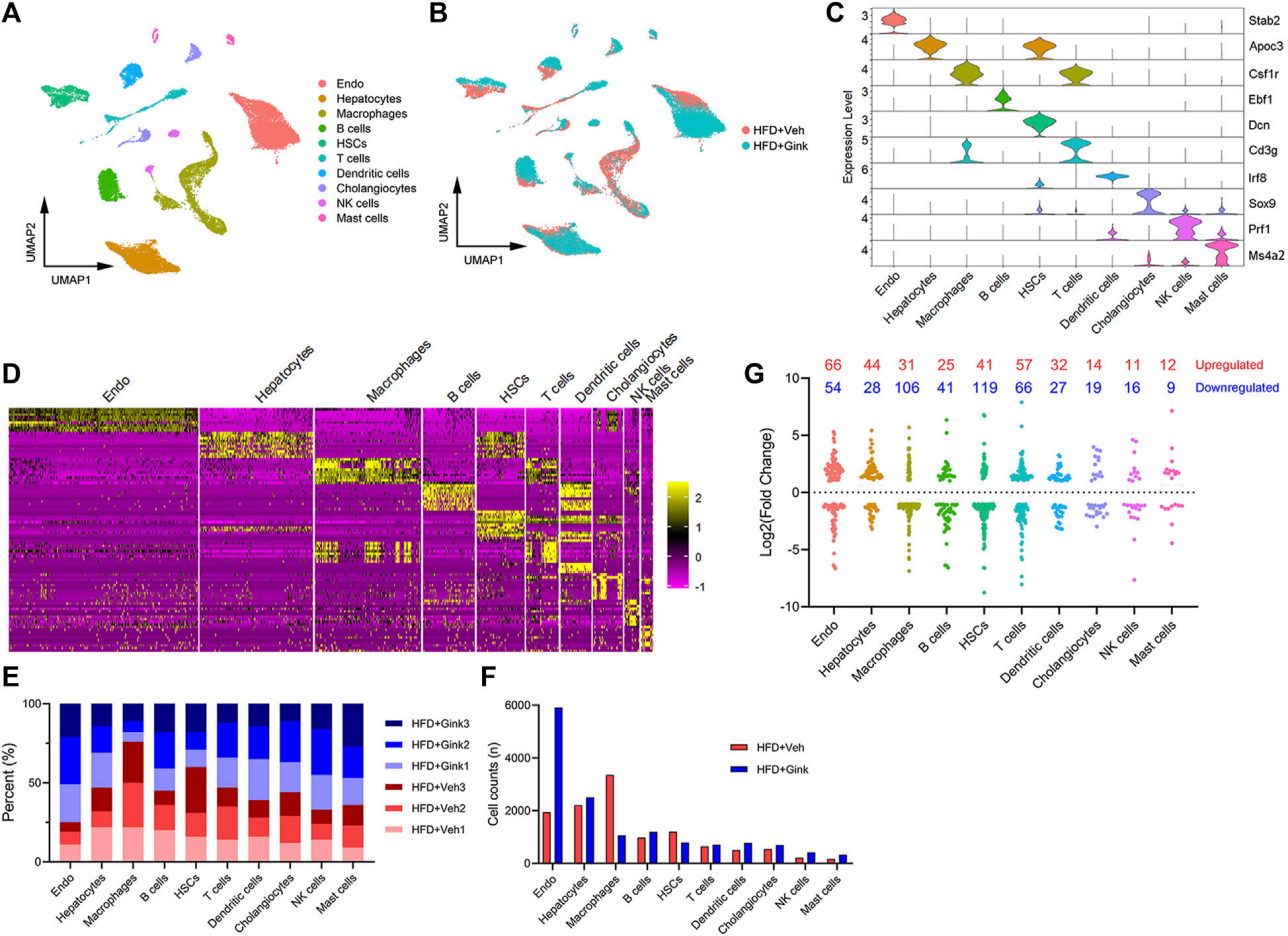
scRNA-Seq analysis of liver cells from HFD + Veh or HFD + Gink groups. **(A)** UMAP plot revealed that the liver cells were divided into 10 clusters based on marker genes. **(B)** UMAP plot for liver cells from HFD + Veh and HFD + Gink groups. **(C)** marker gene expression for the 10 clusters shown by violin plots. **(D)** Top 10 marker genes of the 10 clusters shown by heatmap. **(E)** Cell percent of the 10 clusters in each sample. **(F)** Cell counts of the 10 clusters in the two groups. **(G)** Changes in gene expression by ginkgetin in the 10 clusters between HFD + Veh and HFD + Gink groups.

scRNA-seq analysis was conducted to explore the cellular heterogeneity in LNPCs isolated from NASH mice treated with vehicle or ginkgetin (*n* = 3), which are essential in regulating the process of NASH**.** After quality control filtering, transcriptomes of 26,160 cells were obtained, including 11,754 cells from vehicle-treated mice and 14,406 from ginkgetin-treated mice. And UMAP dimensionality reduction analysis revealed that the cells were divided into ten clusters according to the marker genes, including endothelial cells, hepatocytes, macrophages, B cells, hepatic stellate cells (HSCs), T-cells, dendritic cells, cholangiocytes, NK cells, and Mast cells (Figures 4A–D). And the cell counts and percent were presented (Figures 4E, F). Remarkably, we observed that endothelial cells and macrophages were the most abundant clusters of LNPCs, accounting for 57.2% of the LNPCs. And 75.3% of endothelial cells were from ginkgetin-treated mice, whereas 76.0% of macrophages were from vehicle-treated mice. Additionally, ginkgetin-induced alteration in gene expression was mainly reflected in macrophages and HSCs with a marked reduction (Figure 4G). These data suggested that the impacts of ginkgetin on NASH may be associated with the reprogramming of macrophages, HSCs, and endothelial cells.

### Effects of ginkgetin on hepatic macrophages in NASH mice

**FIGURE 5:**
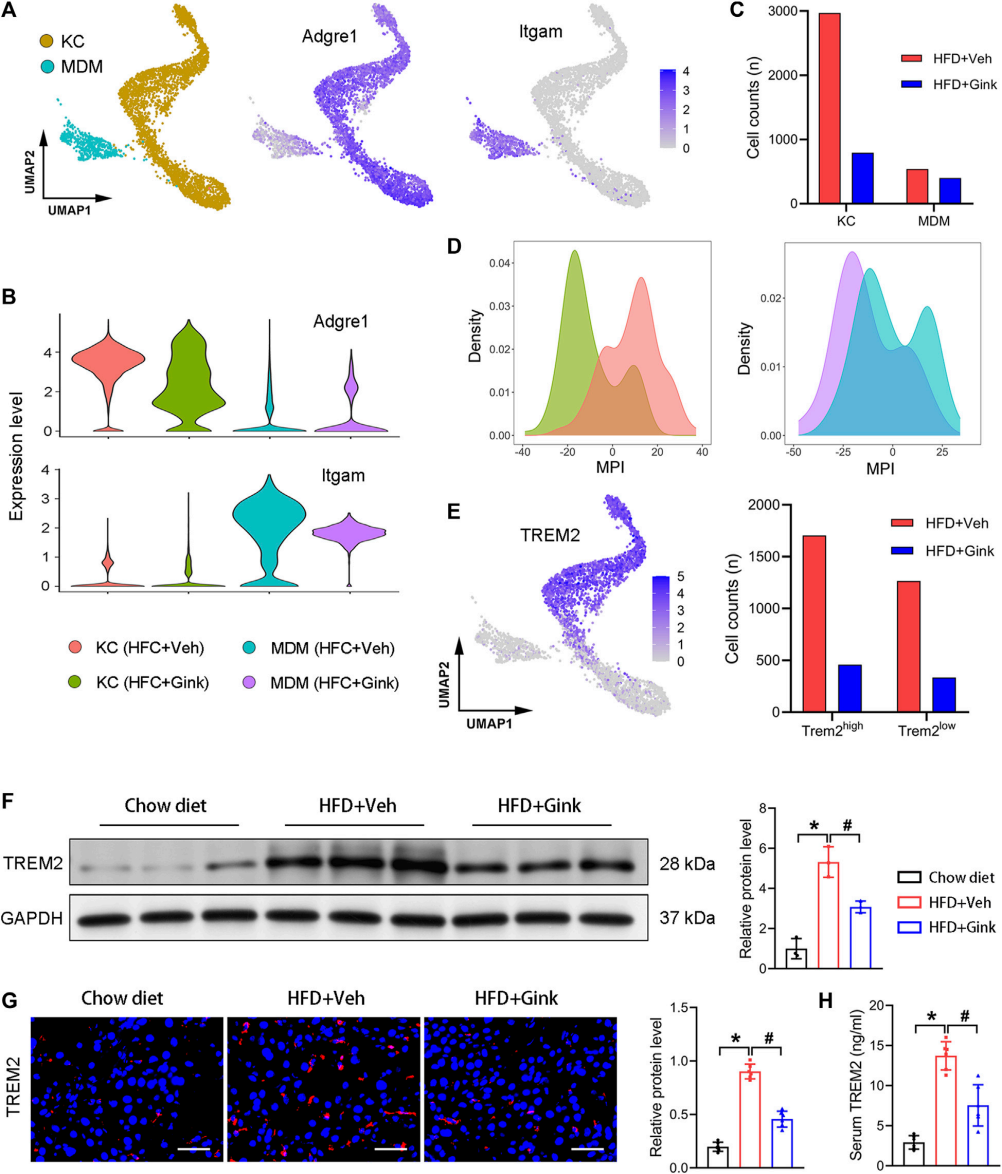
Effects of ginkgetin on hepatic macrophages in NASH mice. **(A)** Kupffer cells (KCs) and monocyte-derived macrophages (MDMs) and their marker genes shown by UMAP plot. **(B)** Marker gene expression of KCs and MDMs shown by violin plots. **(C)** Cell counts of KCs and MDMs in HFD + Veh and HFD + Gink groups. **(D)** MPI of KCs and MDMs in the two groups. **(E)** Subcluster of Trem2 high expression macrophage. **(F)** Western blot analysis of TREM2 in the chow diet, HFD + Veh, and HFD + Gink groups (*n* = 3). **(G)** TREM2 immunofluorescence of liver tissues from the three groups (*n* = 6). Scale bar = 50 μm. **(H)** Levels of serum TREM2 in the three groups (*n* = 6). *: *p* < 0.05, chow diet vs. HFD + Veh groups. #: *p* < 0.05, HFD + Veh vs. HFD + Gink groups.

4,419 macrophages were retained in scRNA-Seq analysis, which were further divided into two subclusters, including Kupffer cells (KCs, high expression of Adgre1) and monocyte-derived macrophages (MDMs, high expression of Itgam) (Figures 5A, B). And ginkgetin induced a significant reduction in macrophages, especially in KCs (Figure 5C). In addition, we also assessed macrophage polarization using MPI based on scRNA-Seq. As expected, we observed a marked alteration toward an anti-inflammatory phenotype in both KCs and MDMs after ginkgetin treatment (Figure 5D).

Next, we analyzed the alteration of NASH-associated macrophages (NAMs), which are subcluster of KCs with high expression of Trem2 (25). NAMs emerge during NASH and play a protective role against NASH. And the level of NAMs is positively correlated with the severity of NASH. Consistent with the improvement of lipid accumulation in the liver, NAMs markedly decreased in ginkgetin-treated mice (Figure 5E). Additionally, both Western blot and Immunofluorescence revealed that the level of TREM2 was significantly downregulated by ginkgetin (Figures 5F, G). Meanwhile, we also measured the level of TREM2 in the serum, which is a circulated marker of NASH and has a positive correction with NAMs (Hendrikx et al., 2022). Accordingly, the elevated TREM2 in serum during NASH also decreased after ginkgetin treatment (Figure 5H).

### Effect of ginkgetin on HSCs in NASH mice

**FIGURE 6:**
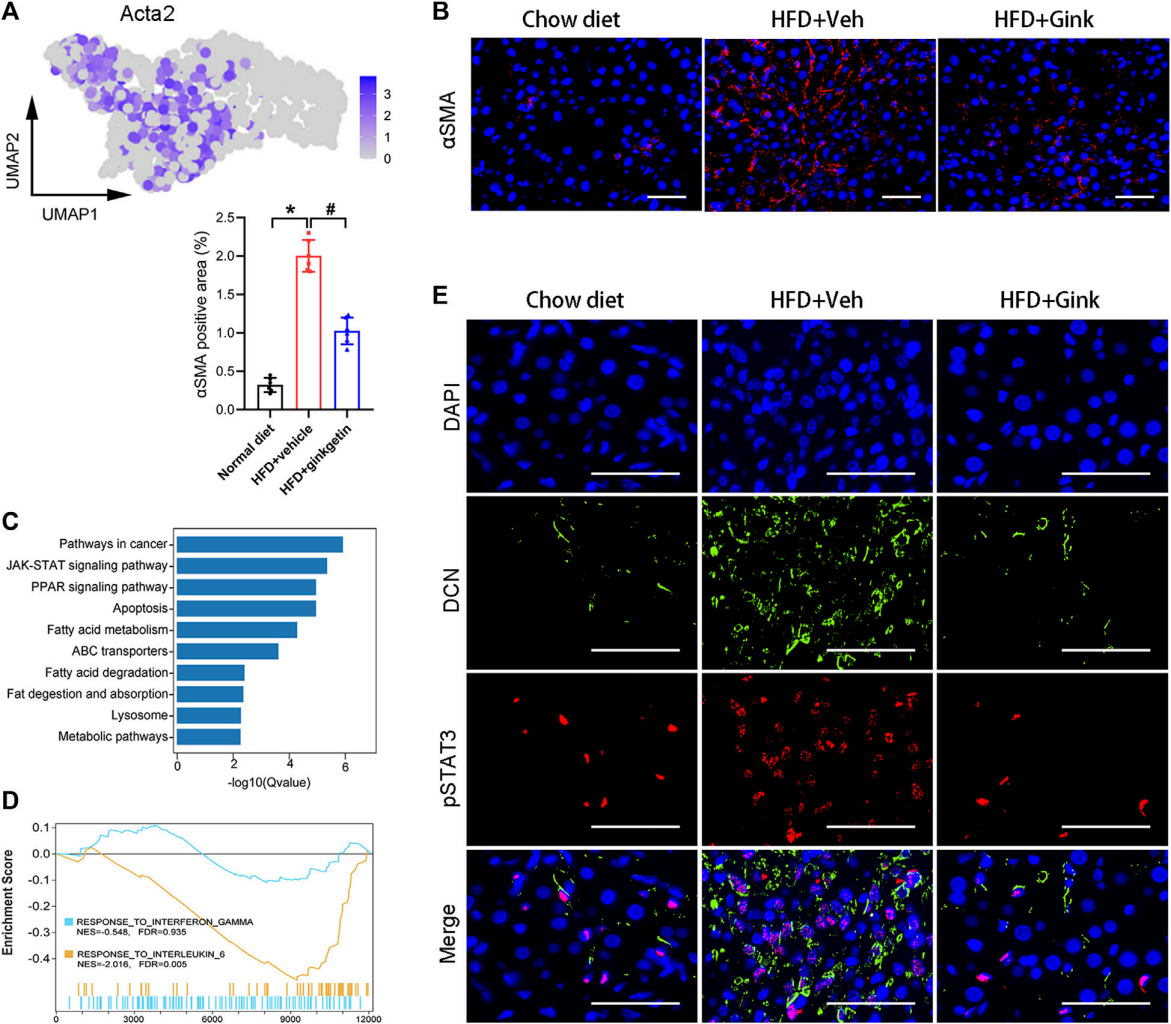
Effects of ginkgetin on hepatic stellate cells (HSCs) in NASH mice. **(A)** Activated HSCs marked by Acta2 shown by UMAP plot. **(B)** αSMA immunofluorescence of liver tissues from chow diet, HFD + Veh, and HFD + Gink groups (*n* = 6). Scale bar = 50 μm. **(C)** KEGG enrichment analysis of DEGs between HFD + Veh and HFD + Gink groups. **(D)** GSEA showing the ginkgetin-induced alteration in IFNγ- and IL6-mediated signaling. **(E)** pSTAT3 and DCN immunofluorescence of liver tissues from chow diet, HFD + Veh, and HFD + Gink groups (*n* = 6). Scale bar = 50 μm *: *p* < 0.05, chow diet vs. HFD + Veh groups. #: *p* < 0.05, HFD + Veh vs. HFD + Gink groups.

HSC activation, marked by high expression of Acta2, is a core step for liver fibrosis (Higashi et al., 2017). scRNA-Seq analysis revealed a significant decrease in the expression of Acta2 in ginkgetin-treated mice (Figure 6A), which was supported by the Immunofluorescence of αSMA (encoded by Acta2) (Figure 6B). Then, we performed KEGG enrichment analysis of the DEGs specially derived from HSCs. In addition to the pathways associated with lipid metabolism, we also discovered that JAK/STAT pathway was enriched (Figure 6C). Increasing evidence has demonstrated that JAK/STAT pathway was essential for HSC activation, especially IL6/STAT3 pathway and IFNγ/STAT1 pathways (Gao et al., 2012; Deng et al., 2013; Su et al., 2015; Xiang et al., 2018; Marti-Rodrigo et al., 2020). Triggering IL6/STAT3 signaling enhances HSC activation and liver fibrosis, while IFNγ/STAT1 signaling exhibits opposite effects. GSEA revealed that ginkgetin induced a marked downregulation in IL6/STAT3 signaling, but no alteration in IFNγ/STAT1 signaling (Figure 6D). Furthermore, immunofluorescence showed an increase in pSTAT3 and DCN (mainly expressed by HSCs) with a close colocation in NASH mice, which was abolished after ginkgetin treatment (Figure 6E). These results suggested that IL6/STAT3 signaling may be associated with ginkgetin-induced inhibition of HSC activation and liver fibrosis.

### Effects of hepatic endothelial cells in NASH mice

**FIGURE 7:**
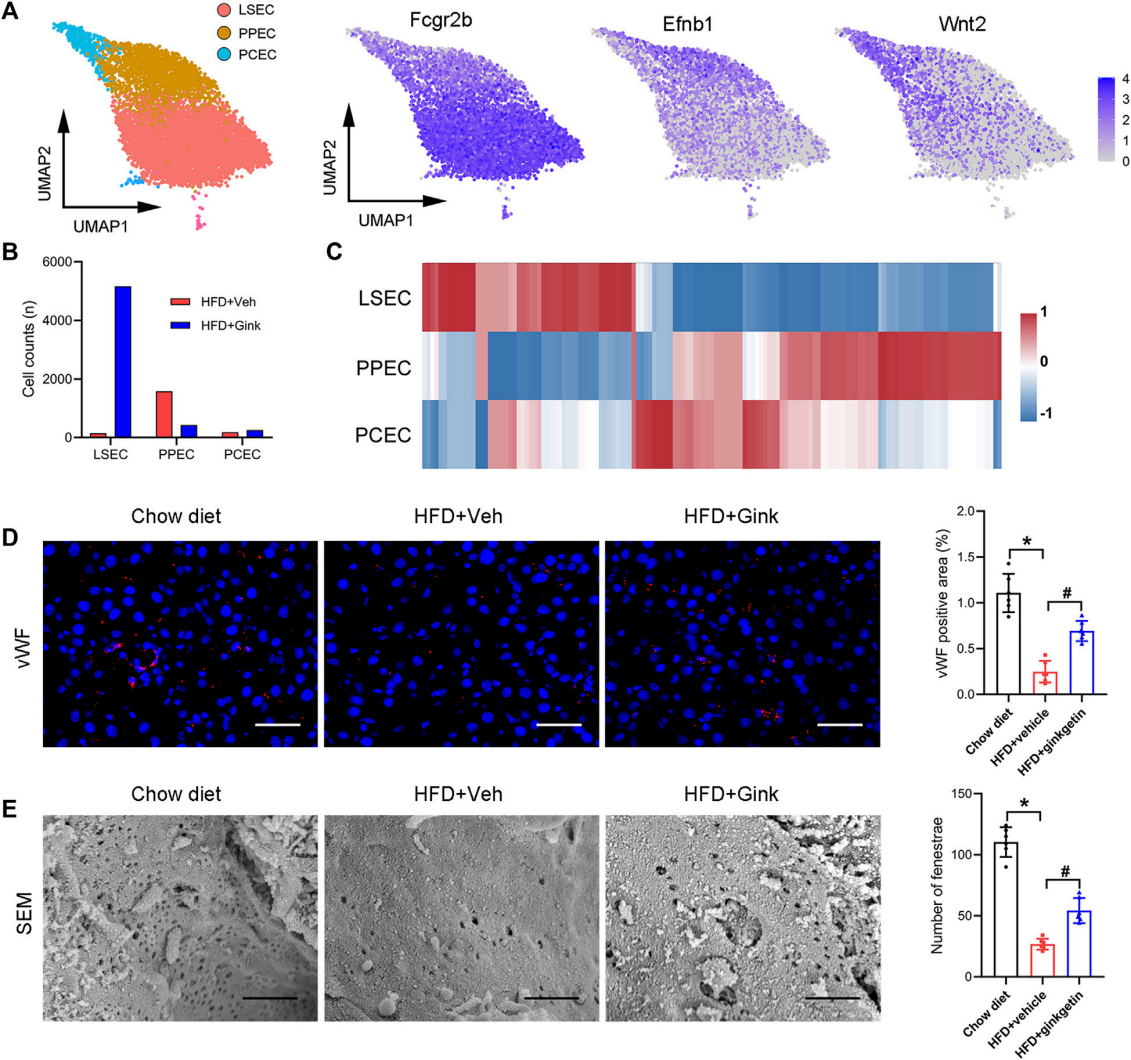
Effects of ginkgetin on hepatic endothelial cells in NASH mice. **(A)** Endothelial cells were divided into three subclusters by marker genes shown by UMAP plot. **(B)** Cell counts of the three subclusters in HFD + Veh and HFD + Gink groups. **(C)** Gene expression in the three subclusters shown by heatmap. **(D)** vWF immunofluorescence of liver tissues from chow diet, HFD + Veh, and HFD + Gink groups (*n* = 6). Scale bar = 50 μm. **(E)** SEM analysis of fenestrae in LSECs (*n* = 6). Scale bar = 1 μm *: *p* < 0.05, chow diet vs. HFD + Veh groups. #: *p* < 0.05, HFD + Veh vs. HFD + Gink groups.

Endothelial cells make up the largest cluster of LNPCs, which can be further divided into three subclusters, including liver sinusoidal endothelial cells (LSECs, high expression of Fcgr2b), per-portal endothelial cells (PPECs, high expression of Efnb1), and peri-central endothelial cells (PCECs, high expression of Wnt2) (Figure 7A). Cluster analysis revealed individual transcriptomic features in the three subclusters (Figure 7B). And in vehicle-treated mice, the main type of endothelial cells was PPECs, while LSECs became the dominant type after ginkgetin treatment (Figure 7C). Consistently, immunofluorescence of vWF showed that NASH resulted in a marked decrease of endothelial cells, which was restored to the normal level after ginkgetin treatment (Figure 7D). Furthermore, SEM analysis revealed that the number of LSEC fenestrae markedly reduced due to NASH, but increased in ginkgetin-treated mice (Figure 7E).

## Discussion

In this study, we demonstrated that ginkgetin exhibits beneficial effects on NASH, including reducing lipid accumulation and inhibiting hepatic inflammation and fibrosis. And these results were supported by bulk RNA-Seq analysis, in which the related signaling pathways and gene expression were markedly downregulated. Furthermore, we assessed the alteration in gene profile by scRNA-Seq, which further uncovered the mechanism of ginkgetin-induced NASH alleviation.

Previous studies have demonstrated that excessive lipid accumulation in hepatocytes is the primary and driving factor for NASH progression by triggering oxidative stress and releasing inflammatory cytokines (Schuster et al., 2018; Haas et al., 2019). And the cytokines enhance inflammatory cell infiltration and hepatocellular injury. Remarkably, ginkgetin induced a clear decrease in lipid accumulation in hepatocytes with improved liver function, which was supported by the downregulation of gene expression and signaling pathways associated with lipid metabolism. These exciting results urged us to explore the involved mechanisms, especially focusing on LNPCs, which are essential in regulating the process of NASH(Xiong et al., 2019).

Among LNPCs, macrophages occupy a central place in NASH pathogenesis and have been considered potential therapeutic targets. And macrophages exhibit strong heterogeneity in performing various complex functions. In this study, scRNA-Seq analysis showed that macrophages not only markedly decreased in number but also shifted from pro-inflammatory to anti-inflammatory phenotype, which was supported by the histological study and RNA-Seq analysis. We also pay attention to NAMs, which are present during NASH and characterized by high expression of Trem2. Recent studies indicated that NAMs play a protective role against NASH by enhancing NAM-dependent efferocytosis of apoptotic hepatocytes caused by lipid overload (Hou et al., 2021; Hendrikx et al., 2022; Wang et al., 2023). And the level of NAMs is positively correlated with the severity of NASH. Consistent with the improvement of lipid accumulation in the liver, ginkgetin induced a marked decrease in NAMs, evidenced by scRNA-Seq, Western blot, and immunofluorescence. Further studies are necessary to investigate whether the impacts on macrophages are directly caused by ginkgetin or secondary to other effects.

NASH has a high risk of developing fibrosis by activating HSCs (Schuppan et al., 2018). In this study, liver fibrosis caused by NASH was markedly suppressed by ginkgetin. Moreover, we observed that the downregulation of IL6/STAT3 signaling was involved in the ginkgetin-induced inhibition of HSC activation. Previous studies have also suggested that IL6/STAT3 signaling was a promising target for treating liver fibrosis (Deng et al., 2013; Su et al., 2015; Xiang et al., 2018; Marti-Rodrigo et al., 2020). Thus, our study could potentially provide a therapeutic strategy for chronic liver fibrosis.

Finally, we assessed the impacts of ginkgetin on endothelial cells in NASH mice. Unlike the anti-angiogenesis mechanism in treating liver fibrosis (Gao et al., 2016; Winkler et al., 2021), our data indicated that ginkgetin promotes hepatic angiogenesis and endothelial cell proliferation. We speculated that the reasons might be as follows: hepatic angiogenesis is a compensatory mechanism of portal hypertension in liver fibrosis, and in turn, angiogenesis promotes fibrosis (Parola and Pinzani, 2019; Gracia-Sancho et al., 2021). Therefore, it is effective to treat liver fibrosis by inhibiting angiogenesis. Contrary to liver fibrosis, hepatic angiogenesis decreases during NASH due to the damage of endothelial cells by abnormal lipid metabolism. Thus, the recovery of endothelial cells after ginkgetin treatment might be secondary to the reduced lipid accumulation in the liver. Moreover, ginkgetin induced a marked increase in LSEC fenestrae, which promoted the substance exchange between hepatocytes and portal vein.

In conclusion, this study provided evidence that ginkgetin ameliorates NASH with a unique perspective at bulk and single-cell levels. These data may promote pharmacological therapy for NASH and raise the existent understanding of NASH.

## Figures and Tables

**Table:** 

**Acta2**	Actin alpha 2
**Adgre1**	Adhesion G protein-coupled receptor E1
**ALT**	Alanine aminotransferase
**ARE**	Androgen response elements
**AST**	Aspartate aminotransferase
**cDNA**	Complementary DNA
**CEBPα**	Recombinant CCAAT/Enhancer Binding Protein Alpha
**COL1A1**	Collagen Type I Alpha 1
**CST**	Cell signaling technology
**DAPI**	4,6-diamino-2-phenyl indole
**DCN**	Decorin
**DEG**	Differently expressed gene
**DMSO**	Dimethyl sulfoxide
**ECM**	Extracellular matrix
**Efnb1**	Ephrin-B1
**ELISA**	Enzyme-linked immunospecific assay
**FASN**	Fatty acid synthase
**FBW**	Fasting body weight
**FC**	Fold change
**Fcgr2b**	Fc fragment of IgG receptor IIb
**FDR**	False discovery rate
**GAPDH**	Glyceraldehyde-3-phosphate dehydrogenase
**GEO**	Gene expression omnibus
**GSEA**	Gene set enrichment analysis
**HE**	Hematoxylin-eosin
**HFD**	High-fat diet
**HSC**	Hepatic stellate cell
**IL**	Interleukin
**Itgam**	Integrin subunit alpha M
**JAK**	Janus kinase
**KC**	Kupffer cell
**KEGG**	Kyoto encyclopedia of genes and genomes
**LNPC**	Liver non-parenchymal cell
**LPS**	Lipopolysaccharide
**LSEC**	Liver sinusoidal endothelial cell
**LW**	Liver weight
**MDM**	Monocyte-derived macrophage
**MPI**	Macrophage polarization index
**NAFLD**	Nonalcoholic fatty liver disease
**NAM**	NASH-associated macrophage
**NAS**	NAFLD activity score
**NASH**	Nonalcoholic steatohepatitis
**NF-κB**	Nuclear factor kappa-B
**NIH**	National Institutes of Health
**NK**	Natural killer cell
**Nrf2**	Nuclear factor erythroid 2-related factor 2
**PCA**	Principal component analysis
**PCEC**	Peri-central endothelial cell
**PPARγ**	Peroxisome proliferator-activated receptor gamma
**PPEC**	Per-portal endothelial cell
**PVDF**	Polyvinylidene fluoride
**RIPA**	Radio immunoprecipitation assay
**scRNA-Seq**	Single-cell RNA sequencing
**SEM**	Scanning electron microscopy
**SMA**	Smooth muscle actin
**STAT**	Signal transducer and activator of transcription
**TNFα**	Tumor necrosis factor alpha
**Trem2**	Triggering receptor expressed on myeloid cells-2
**UMAP**	Uniform manifold approximation and projection
**UMI**	Unique molecular identifier
**vWF**	Von Willebrand factor

## Data Availability

The datasets presented in this study can be found in online repositories. The names of the repository/repositories and accession number(s) can be found below: NCBI Gene Expression Omnibus (GEO) (https://www.ncbi.nlm.nih.gov/geo/), GSE235939 and GSE235797.
