# Supplementary material for: Beneficial effects of ginkgetin on improving nonalcoholic steatohepatitis characterized by bulk and single-cell RNA sequencing analysis
Source: Front Pharmacol. 2023 Oct 4;14:1267445. doi: 10.3389/fphar.2023.1267445 (PMC10582714; doi:10.3389/fphar.2023.1267445)
Supplement: Supplementary file 2 [file Table1.DOCX]

**Supplementary Table 1. Antibody list**

| **Antibodies** | **Company** | **Dilution** |
| --- | --- | --- |
| Anti-FASN | Abcam (ab128856) | 1:1000 for western blot |
| Anti-PPARγ | Abcam (ab272718) | 1:1000 for western blot |
| Anti-F4/80 | Abcam (ab300421) | 1:50 for immunofluorescence |
| Anti-TNFα | Servicebio (GB11188-100) | 1:500 for western blot |
| Anti-NF-κB p65 | Cell Signaling Technology (8242) | 1:1000 for western blot |
| Anti-p-NF-κB p65 (Ser536) | Cell Signaling Technology (3033) | 1:1000 for western blot |
| Anti-COL1A1 | Cell Signaling Technology (72026) | 1:1000 for western blot |
| Anti-TREM2 | Abcam (ab305103) | 1:1000 for western blot  1:500 for immunofluorescence |
| Anti-αSMA | Cell Signaling Technology (19245) | 1:500 for immunofluorescence |
| Anti-DCN | Abcam (ab175404) | 1:100 for immunofluorescence |
| Anti-pSTAT3 (Try705) | Cell Signaling Technology (9145) | 1:100 for immunofluorescence |
| Anti-vWF | Abcam (ab287962) | 1:50 for immunofluorescence |
| Anti-GAPDH | Cell Signaling Technology (5174) | 1:1000 for western blot |
